# Explanation of Obsessive-Compulsive Disorder and Major Depressive Disorder on the Basis of Thought-Action Fusion

**Published:** 2013

**Authors:** Hossein Ghamari Kivi, Ne’mat Mohammadipour Rik, Fariba Sadeghi Movahhed

**Affiliations:** 1 Department of Psychology, School of Literature and Humanistic Science, University of Mohaghegh Ardabili, Ardabil, Iran.; 2 Assistant Professor, Department of Psychiatry, School of Medicine, Ardabil University of Medical Sciences. Ardabil province, Iran

**Keywords:** Major Depressive Disorder, Obsessive-Compulsive Disorder, Thought-Action Fusion (TAF)

## Abstract

**Objective**: Thought-action fusion (TAF) refers to the tendency to assume incorrect causal relationship between one’s own thoughts and external reality, in which, thoughts and actions are treated as equivalents. This construct is present to development and maintenance of many psychological disorders. The aim of the present study was to predict obsessive-compulsive disorder (OCD) and its types, and major depressive disorder (MDD) with TAF and its levels.

**Methods:** Two groups, included 50 persons with OCD and MDD, respectively, were selected by convenience sampling method in private and governmental psychiatric centers in Ardabil, Iran. Then, they responded to Beck Depression Inventory, Padua Inventory and TAF scale. Data were analysed using multiple regressions analysis by stepwise method.

**Results: **TAF or its subtypes (moral TAF, likelihood-self TAF and likelihood-others TAF) can explain 14% of MDD variance (p < 0.01), 15% of OCD variance (p < 0.01), and 8-21% of OCD types variance (p < 0.05). Moral TAF had high levels in OCD and MDD.

**Conclusion**: The construct of TAF is not specific factor for OCD, and it is present in MDD, too.

**Declaration of interest:** None.

## Introduction

Thought-action fusion (TAF) refers to the belief that thoughts and actions are inextricably linked (1). In the TAF theory, thought and action are treated as equivalents (2). The contemporary TAF concept arose from Rachman’s (3) and Salkovskis’ (4) theories and clinical observations of patients with obsessional thinking, where it was noticed that the patients assume a thought like an action (4). 

Shafran et al. (5), first formally introduced and investigated the concept. They developed a measure of TAF that has been incorporated into most subsequent researches. TAF has two forms: moral TAF which is the belief that unacceptable thoughts are morally equivalent to overt unacceptable actions; and likelihood TAF which refers to the belief that certain thoughts cause particular events, or at least increase the likelihood of such events occurring. Two domains of likelihood TAF have been proposed: A: likelihood self, which refers to events occurring to oneself, and B: likelihood others, which refers to events occurring to others, as a consequence of one’s thoughts (1).

TAF concept has compiled since the 1990s in OCD articles. Before, the researchers studied this concept as magical thinking (5). Magical thinking refers to beliefs that defy culturally accepted laws of causality (6). It has been argued to be a central cognitive feature of OCD (6, 7).

Two major disorders that are widely associated with TAF are OCD and major depressive disorder (MDD). OCD is defined by two central phenomena: obsessions (i.e., persistent ideas, thoughts, impulses, or images that are experienced as intrusive and inappropriate and that cause marked anxiety or distress) and compulsions as repetitive behaviors (e.g., hand washing, ordering, or checking) or mental acts (e.g., praying, counting, or repeating words silently) the goal of which is to prevent or reduce anxiety or distress, not to provide pleasure or gratification (8). Most obsessions involve thoughts about contamination, repeated doubts, a need to have things in a particular order, aggressive impulses, or sexual imagery, and the most common compulsions involve cleaning and checking (9).

TAF is one of a number of cognitive variables that have been extensively researched in relation to OCD and other anxiety disorders in recent years. The impetus for the increasing attention paid to cognitive constructs in OCD has been dissatisfaction with the traditional concept of OCD as a condition in which compulsions develop with the main purpose of alleviating anxiety. Clinical experience and research have repeatedly indicated that underlying beliefs and appraisals are often intervening factors between obsessions and compulsions and those they often play a role in maintaining OCD (1).

Review of studies about TAF shows that TAF is one of the fundamental cognitive bases which plays a fundamental role in the development and maintenance of OCD (9-18).

Average correlations between total scores on the Thought-Action Fusion Scale (TAFS) (5) and the Maudsley Obsessive-Compulsive Inventory (MOCI) (10) have been consistently found (between 0.20 and 0.38) (9, 11-16). A similar magnitude of relationship appears to exist for the association between TAF scale scores and the Padua (17) and Padua revised (18) scales as well (6, 11, 14). Correlations between each TAF subscale and obsessive-compulsive symptoms also appear to fall within this range (5, 12, 14).

Previous studies have found TAF related to not only OCD, but also to other disorders such as MDD, panic, generalized anxiety disorder, social phobia (19-21), schizophrenia (22), schizotypy (23), and also variety features such as inflated sense of responsibility and thought suppression (15, 16, 24), intrusion (25), and guilt (9, 26).

TAF appears to be related to depression in both adolescents and adults. However, the significant correlations between TAF and depression have typically been small to medium in magnitude [r = 0.42 in an adolescent sample (19), r = 0.33 (21), r = 0.38 (26), r = 0.15 (14), and r = 0.10 to 0.42 (5)]. 

Shafran et al. (5), and Rassin et al. (12, 14) have shown that TAF-likelihood was more strongly associated with obsessionality than TAF-morality, and depression was more related to TAF-morality. Abramowitz et al. (19) found that only TAF moral (r = 0.22), but not TAF-likelihood-other (r = - 0.05) or TAF-likelihood-self (r = - 0.05) were correlated with depression. These findings suggest that TAF-moral may be more directly related to depressive symptoms than TAF-likelihood, which is perhaps more related to anxiety. In contrast to Shafran et al. (5), and Rassin et al. (12, 14), Yorulmaz et al. (9) in Turkish samples suggested as compared to TAF-likelihood, TAF-morality scores seemed to be more strongly correlated with obsessive-compulsive symptoms. They attributed these differences to cultural differences and religious beliefs of their sample. 

A research on OCD reported that between symptoms of OCD and TAF, a positive and significant correlation exists, and sub-scale of likelihood-other TAF is the best predictor of compulsions and the cluster of checking symptoms and sub-scale of likelihood-self TAF is the best predictor of obsessions and the cluster of doubting symptoms (27). Another study showed that between TAF beliefs and the symptoms of OCD a significant positive relationship exists (28). Also, another study in Shiraz, Iran, studied students and reported that subscales of TAF predicted the main symptoms of OCD (29).

TAF is assumed to be one of the cognitive components involved in MDD (9, 12, 19, 21) as well as OCD (5, 7, 12, 14). Zucker et al. (30) noted that even short and simple educational interventions (providing simple messages of anti-TAF) can be effective in treatment of MDD and OCD. 

Considering that researches done in this area does not clearly express relationship of TAF and its various types to psychopathology such as OCD and MDD, and on the other hand, there is very few published reports in our country with subject of TAF, this study carried out to answer to two questions:

1- How much is the share of each type of TAF in prediction of MDD? 

2- How much is the share of each type of TAF in prediction of OCD and its variants?

## Materials and Methods

***Subjects***

Fifty patients with OCD (10 men and 40 women) and 50 patients with MDD (8 men and 42 women) were gathered by convenience sampling method in private and governmental psychiatric centers in Ardabil, Iran, in 2010. These patients were referred to psychiatrists and had received the diagnosis of MDD or OCD by them. The disease duration was from 1 to 3 years and the age ranged from 16 to 38 years. Inclusion criteria were not receiving any psychiatric medicines in the past, first referral to psychiatrist and being at least at the level of guidance school. Written consent was obtained from the subjects.

***Procedure***

First, the participants were interviewed based on the criteria of DSM-IV-TR clinical interviews by clinical psychologists to confirm the diagnosis. Then, the patients with OCD responded to Padua Inventory and TAF scale and patients with MDD responded to Beck Depression Inventory (BDI) and TAF scale. The questionnaires were completed by the participants in psychiatrists' offices. 

After data collection, data were analyzed by the SPSS software for Windows (version 16.0, SPSS Inc., Chicago, IL, USA) using multiple regressions analysis by stepwise method.

***Measures***

The following self-report measures were completed by the subjects: 

*Thought-action fusion scale (TAFS)* (5): 

This is a 19-item self-report measure of the tendency to fuse thoughts and actions. It contains 12 items that assess moral TAF (e.g., “Having a blasphemous thought is almost as sinful to me as a blasphemous action”); 3 items that assess likelihood-self TAF (e.g., “If I think of myself being in a car accident, this increases the risk that I will have a car accident”); and 4 items that assess likelihood-other TAF (e.g., “If I think of a relative/friend losing their job, this increases the risk that they will lose their job). Each item is rated on a scale from 0 (strongly disagree) to 4 (strongly agree). The instrument’s psychometric properties have been described by Shafran et al. (5) and Yorulmaz et al. (9) between 0.85 and 0.96. 

Subsequent to translating the questionnaire to Persian and its back translation to original language, a pilot study based on cultural differences was carried out on thirty students. In this study, Cronbach's alpha coefficient was obtained as 0.82.

*Padua Inventory-Washington State University Revision (PI-WSUR)* (31):

This inventory is revision of the original Padua inventory (17) that included 39 items and 5 subscales. This inventory is a self-report measure designed by Burns et al. (31). The instrument provides 5 sub-scales: contamination obsessions and washing compulsions (COWC), dressing/grooming compulsions (DRGRC), checking compulsions (CHCK), obsessional thoughts of harm to self/others (OTHSO), and obsessional impulses to harm self/others (OIHSO). All items are scored on a 0 (not at all) to 4 (very much) scale with a total score range of 0-156. Scores for the 5 sub-scales are calculated by summing the appropriate items. 

MacDonald and De Silva (32) reported Padua reliability coefficient between 0.76 to 0.96 and its internal consistency as 0.96. Marino et al. (24) found a Cronbach’s alpha coefficient of 0.93 for the Padua inventory. Shams et al. (33), in an Iranian sample, reported its Cronbach’s alpha as 0.92 with Spearman split of 0.95 and test-retest (r = 0.77).

*Beck Depression Inventory (BDI)*:

This is a 21-item measure that is widely used to assess somatic, affective, and behavioral symptoms of depression (34). Scores range from 0 (no symptoms) to 63 (very severe symptoms). The sound psychometric properties of the scale are supported by an extensive literature (35). It has been reported to have a Cronbach's alpha of 0.87 in an Iranian sample (36). 

## Results

Mean and standard deviations (SD) of age, TAF subscales and BDI score in patients with MDD are presented in table 1. Those of patients with OCD are presented in table 2.

**Table 1:** Mean and standard deviation (SD) of variables in the Major depressive disorder group

**Variables**	**Mean (SD)**
Age	26.40 (5.80)
TAFt	45.62 (1.00)
TAFm	31.62 (8.53)
TAFLs	7.50 (2.56)
TAFLo	7.46 (3.39)
BDI	33.10 (9.49)

TAF: Thought-action fusion, TAFt: Total TAF, TAFm: Moral TAF, TAFLs: Likelihood-self TAF, TAFLo: Likelihood-others TAF, BDI: Beck Depression Inventory

**Table 2:** Mean and standard deviation (SD) of variables in the obsessive-compulsive disorder group

**Variables**	**Mean (SD)**
Age	29.80 (8.10)
TAFt	45.40 (1.00)
TAFm	30.69 (8.36)
TAFLs	7.40 (3.03)
TAFLo	7.37 (4.31)
Total score of Padua inventory	72.92 (1.95)
COWC	23.60 (9.82)
DRGRC	6.84 (3.30)
CHCK	24.44 (7.65)
OTHSO	12.66 (5.51)
OIHSO	5.82 (4.33)

TAF: Thought-action fusion, TAFt: Total TAF, TAFm: Moral TAF, TAFLs: Likelihood-self TAF, TAFLo: Llikelihood-others TAF, COWC: Sub-group of contamination obsessions and washing compulsions of OCD group, DRGRC: Sub-group of dressing/grooming compulsions of OCD group, CHCK: Sub-group of compulsions checking of OCD group, OTHSO: Sub-group of obsessional thoughts of harm to self/others of OCD group, OIHSO: Sub-group of obsessional impulses to harm self/others of OCD group

Table 3 shows the results of multivariate regression analysis with stepwise method. Total TAF, moral TAF, likelihood-self TAF and likelihood-others TAF were entered to the analysis as predictor variables. 

Results suggested that moral TAF can explain 14% of MDD variance (p < 0.01), 15% of OCD variance (p < 0.01), 8% of sub-group of contamination obsessions and washing compulsions of OCD group variance (p < 0.05), and 11% of sub-group of compulsions checking of OCD group variance (p < 0.05). 

Likelihood-self TAF could predict 9% of sub-group of dressing/grooming compulsions of OCD group (p < 0.05), and total TAF could predict 21% of sub-group of obsessional thoughts of harm to self/others of OCD group (p < 0.01). 

In this study, TAF and its subtypes (moral TAF, likelihood-self TAF and likelihood-others TAF) did not explain a special variance in sub-group of obsessional impulses to harm self/others in OCD group. 

## Discussion

Obtained results showed that only moral TAF can be a good explainer for MDD. This is in consistent with former studies (5, 12). They reported that moral TAF in comparison to likelihood TAF was further associated with depression. 

Abramowitz et al. (19) also reported that only moral TAF (not likelihood TAF) was related to depression. Of course, in their study, depression has been studied in general (not just major depressive disorder).

Since moral TAF might be related to self-blame, personalization and guilt tendencies that are evident in depression, it seems that its relationship with depression is higher, because also in depression, self-blame, personalization and guilt are active.

**Table 3:** Summary of results of multivariate regression analysis with stepwise method

**Groups**	**Prediction Variable**	**Dependent Variable**	**R**	**Adjusted R Squares**	**F**	**P**	**Beta**
MDD	Moral TAF	MD	0.40	0.14	09.34	0.004	00.40
OCD	Moral TAF	OCD	0.41	0.15	09.84	0.003	00.41
COWC	Moral TAF	COWC	0.32	0.08	05.54	0.023	00.32
DRGRC	likelihood self TAF	DRGRC	0.33	0.09	06.05	0.018	- 0.33
CHCK	Moral TAF	CHCK	0.36	0.11	07.13	0.010	00.36
OTHSO	Total TAF	OTHSO	0.48	0.21	14.48	0.001	00.48
OIHSO	---	OIHSO	---	---	---	---	---

MDD: Major depressive Disorder, OCD: Obsessive-Compulsive Disorder, COWC: Sub-group of contamination obsessions and washing compulsions of OCD group, DRGRC: Sub-group of dressing/grooming compulsions of OCD group, CHCK: Sub-group of compulsions checking of OCD group, OTHSO: Sub-group of obsessional thoughts of harm to self/others of OCD group, OIHSO: Sub-group of obsessional impulses to harm self/others of OCD group

The results of this study showed that in OCD group, moral TAF can explain about 15% of OCD variance, 8% of sub-group of contamination obsessions and washing compulsions of OCD group variance, and 11% of sub-group of compulsions checking of OCD group variance.

These findings are consistent with a former study in Turkey (9) as well as some Iranian studies (27-29), in which, moral TAF, compared to likelihood TAF, showed more significant correlation with OCD. However, the obtained findings are in contrast to the findings of some studies (5, 14) where correlation between OCD and likelihood TAF was mentioned as low to moderate.

Besides, this study found no significant correlation between sub-groups of obsessional impulses to harm self/others of OCD group with any kind of TAF. This result is in consistent with findings of Emmelkamp and Aardena (32). They reported that TAF was only weakly related to impulses (r = 0.11). Also, this result is disparate with findings of Rachman (3) study, because in her study, TAF showed stronger correlation with impulses than obsessions. This indicates that unlike the results of Western research (5, 14, 15), and to consistent with research of Yorulmaz et al. (9), moral TAF has further correlation with MDD, OCD and its kinds.

Research of Yorulmaz et al. (37), in Turkey showed that Muslim participants are more strongly endorsed the importance of thoughts and of controlling them than did Christians. Islam is a more ritualistic religion in which there are pre-defined behavioral requisites. In addition to faith, believers strive for salvation by following these rules and rituals. For instance, cleanliness, purity and regular prayers that adhere to strict religious rules are important issues in Islam (36). 
